# Editorial: Cognitive Control of Emotions in Challenging Contexts

**DOI:** 10.3389/fnbeh.2021.785875

**Published:** 2021-11-02

**Authors:** Nils Kohn, Carmen Morawetz, Mathias Weymar, Jiajin Yuan, Florin Dolcos

**Affiliations:** ^1^Donders Institute for Brain, Cognition and Behavior, Radboud University Medical Center, Nijmengen, Netherlands; ^2^Faculty of Psychology and Sport Science, Institute for Psychology, University of Innsbruck, Innsbruck, Austria; ^3^Department of Biological Psychology and Affective Science, Faculty of Human Sciences, University of Potsdam, Potsdam, Germany; ^4^Faculty of Health Sciences Brandenburg, University of Potsdam, Potsdam, Germany; ^5^The Affect Cognition and Regulation Laboratory (ACRLab), Key Laboratory of Cognition and Personality of Ministry of Education, Faculty of Psychology, Southwest University, Chongqing, China; ^6^Beckman Institute for Advanced Science and Technology, University of Illinois at Urbana-Champaign, Urbana, IL, United States; ^7^Department of Psychology, University of Illinois at Urbana-Champaign, Champaign, IL, United States

**Keywords:** emotion faces, functional neuroimaging, implicit processing, personality, emotion regulation, context, situation, disposition

The ability to cognitively regulate our emotions has emerged as an important moderating factor to multiple forms of psychopathology and human behavior. For this reason, the field of emotion regulation has faced a growing interest and popularity within social, cognitive, and affective neuroscience over the past two decades. Moving from strictly localized “amygdala-centered” concepts and top-down prefrontal control systems to broader interactive network dynamics (Smith and Lane, 2015; Morawetz et al., 2020) has clearly increased our understanding of how emotions can be controlled using a variety of emotion regulation (ER) strategies and analytic approaches (Morawetz et al., 2017). However, so far, research has mainly focused on investigating particular strategies, rarely considering situational and dispositional factors (Doré et al., 2016). By addressing this issue, this Research Topic contributes to the field of situational and dispositional factors influencing ER.

Situational and dispositional factors have the potential to influence the way we perceive and regulate our emotions. Situational factors may include chronic or acute stress, fatigue, hunger, and other temporally dynamic motivational factors, as well as dispositional factors related to personality and temperamental traits, both vices and virtues. The distinction between dispositional and situational factors is, in part, arbitrary and can be subsumed under challenging (or facilitating) contexts that influence emotional regulation. An acute state of hunger or sleep deprivation may make a person less able or willing to engage in regulatory behavior, leading to a host of sub-optimal decision processes.

This Research Topic brings together papers focusing on the contextual factors that can roughly be described by more situational and dispositional aspects and by their interaction. The present collection of manuscripts contributes substantially to the field by bringing together empirical reports, using a broad range of methodological approaches, along with reviews and opinion pieces. Situational and dispositional emotion regulation is elucidated using various human psychophysiological (hemodynamics, electrophysiology), neurostimulation, and behavioral methods. The Research Topics starts out with a discussion of the situational factors on cognitive ER and moves to their influence on more automatic ER, to make the transition to dispositional factors by highlighting examples of efficient ER training. The Research Topic ends with discussion of physiological and clinical factors influencing ER and demonstrates the broad potential impact of ER trainings as well as the need for multi-disciplinary approaches due to complex interactions.

## Situational Influences on Explicit and Implicit Emotion Regulation

This e-book starts off with a section on situational factors with a focus on ER strategies. Haspert et al. studied the influence of acceptance-based regulation of painful stimuli, and found that participants were able to regulate both subjective pain intensity and unpleasantness ratings in acceptance trials. Additionally, heart rate was reduced, which indicates the use of acceptance-based strategies as a potential way of coping with pain. In their meta-analysis, Zaehringer et al. summarize evidence regarding the impact of ER strategies on psychophysiological measures. They find little convergence and only small mean effect sizes of reappraisal and suppression on autonomic measures and medium effect sizes for electromyographic measures. The authors further demonstrate that this inconsistency and surprising lack of effect by standard ER strategies on physiology is brought about by heterogeneities in task design and small sample sizes. This calls for a better standardization of methods in a first step, to better understand the effect of ER strategies on physiology, later on.

The flip side of maladaptive ER strategies is explored by Whiteman and Mangels, who show that rumination (i.e., the tendency to brood over one's problems and feelings) not only has a detrimental effect on mood and mental health (Nolen-Hoeksema et al., 2008; Kohn et al., 2014) but also negatively influences performance on an attention task. Specifically, induction of rumination led to more attention for reminders of errors, compared to corrective information on how to avoid the error in the future. ER strategies have the potential to shift attentional focus away from aversive stimuli (Haspert et al.), but also away from supportive stimuli, highlighting the situational appropriateness of ER strategies (Whiteman and Mangels). The contribution by Zhao et al. moves the focus from internal, situational use of ER strategies to the internalized, but externally focused concept of placebo effects. Placebo effects have characteristic similarities to automatic ER (Braunstein et al., 2017), as it is a top-down regulatory process, but outside of conscious awareness, that instills the belief that a sham treatment (e.g., the placebo) is efficient (Wager and Atlas, 2015). The authors show that a placebo intervention could effectively reduce not only the perception of pain but also empathy for pain and related activity in the posterior insula, hence demonstrating that a placebo mindset has the potential to alter physiology of empathic pain.

Kuehne et al. show that neurostimulation of the dorsolateral prefrontal cortex leads to poorer performance on an automatic ER task—i.e., the face-word Stroop task. This might be related to the detrimental influence of conscious cognitive control stimulated by anodal stimulation during the automatic task, which interferes with efficient task performance (Kuehne et al.). These findings could be taken as an indication of the delicate relationship with cognitive control and the fragility of controlling faculties, which can be influence by many challenging contexts, such as stress (Kohn et al., 2017) of overnight fasting (Kohn et al., 2015). This fragility is also demonstrated by two more papers using a go-nogo task which show that caffeine boosts response related decisions in a sleep deprived state (Chen, Zhang et al.), and that fast paced music interferes with conflict monitoring (Xiao et al.). Emotion control, regardless of whether implicit or explicit ER, necessarily requires the intactness of important cognitive features (Braunstein et al., 2017). Thus, situational interference or facilitation of cognitive abilities will have downstream consequences for eventual attempts to regulate emotion.

## Generation And Influence of Dispositional Factors on Emotion Regulation

Several contributions highlight that dispositional factors do not necessarily have to represent stable and fixed personality characteristics, but preferentially using ER strategies can be a dispositional factor (Garnefski and Kraaij, 2006) and use of ER strategies can be trained (e.g., Dolcos et al.). Dolcos et al. show that training ER strategies can have beneficial effects on cognitive functions. The study impressively demonstrates that ER training can improve resilience and well-being and is reflected in brain and behavior. Furthermore, this influence of training ER strategies on cognition highlights the intertwined nature of cognition and emotion, which influence each other dynamically (Dolcos et al., 2011, 2020; Dolcos and Denkova, 2014). Dolcos et al. also demonstrate that ER training leads to increased connectivity among cognitive and emotion control regions and across regions of self-referential and control networks. Doerfel et al. aimed to replicate studies on the link between habitual use of ER strategies and the amygdala, which underscores the notion that restriction to amygdala connectivity is too reductionistic and ER might rather involve multiple hierarchical networks (Smith and Lane, 2015; Morawetz et al., 2020). Findings by Chen, Yu et al. further indicate that reappraisal via implementation intention technique (Gollwitzer, 1999; Achtziger et al., 2008) might be more efficient in regulating emotions that conscious cognitive regulation, which underlines the huge potential of ER trainings. The review by Panasiti et al. describes how emotion processing and regulation are important factors in Psoriasis, a chronical dermatological condition, which highlights the important interaction of body and emotion and also points to the potentially broad impact of efficient ER trainings. Finally, Wiener et al. describe, for the case of essential hypertonia, how the thalamic pulvinar nucleus might be engaged in the dysregulation of interactions between emotion processing brain networks and attentional/cognitive brain networks, which gives rise to a vicious cycle of negative emotion-physiology interactions.

Moving to concepts closer to stable personality factors and their interaction with ER and the affective and cognitive substrates, Xia et al. demonstrate that individuals with elevated trait anxiety have response inhibition deficits in the go/NoGo task. Interestingly, the authors link the deficits to influences on premotor inhibition control and evaluation and monitoring. This ties into the multi-faceted, hierarchical nature of ER, which relies on multiple brain networks interactions (Smith and Lane, 2015; Morawetz et al., 2020; Dolcos et al.), such as motor and monitoring systems in this study. Demonstrating the interdependence and dynamic nature of dispositional factors in development, Tsai et al. show that the development of anxiety is related to early life stress and mediated by cognitive control abilities in adolescence, with cognitive control having a buffering function for the effect of stress on anxiety. Wagels et al. demonstrate how endogenous testosterone levels influence processing, regulation and expression of angry emotions depending on MAOA polymorphism.

Lischke et al. investigated the interaction of several dispositional factors with biological sex, in essence highlighting the many aspects contributing to the influence of dispositions on ER. The authors found that interoceptive accuracy, as measured by a task in which subjects have to monitor their own heartbeat, was differentially related to habitual use of reappraisal or suppression depending on the biological sex. Specifically, men showed a positive association between reappraisal use and interoceptive success that was absent in women (Lischke et al.). Flores-Torres et al. demonstrate a sex-dependent influence of a humor based mood induction on cognitive performance in the Iowa Gambling task. These findings further emphasize the importance of considering biological sex as a factor in automatic and also cognitive emotion regulation (McRae et al., 2008; Zlomke and Hahn, 2010). Building on findings of the relation of narcissism and emotion regulation (Zhang et al., 2015), Loeffler et al. investigated how facets of narcissism, such as grandiose and vulnerable narcissism, differentially influence emotion regulation abilities, in which sex does not have an influence. They find initial evidence for an increased use of maladaptive ER strategies in vulnerable narcissism, but not grandiose narcissism, which further highlights the need to not only consider multiple networks in the brain, but also consider multiple factors and sub-factors in personality when integrating dispositional effects on ER. This fundamentally calls for a stronger multi-disciplinary collaboration and integration of specific experts in execution and planning of contextual ER studies.

## Conclusions

In summary, this Research Topic explores situational and dispositional factors or contexts that influence ER differentially. Importantly, the contributions highlight the need for a multi-faceted conceptual approach that integrates concepts like stress, fasting, along with trait factors and influence of sex. Given the complex interactive and dynamic nature, we call for an increased multi-disciplinary collaboration of experts in the investigation of contextual ER. At the neural level, integration of multiple, interacting brain networks can be seen as mandatory for future research, which should also more strongly incorporate bi-directional influences of emotion-cognition and emotion-body interactions. These profound interactions lay the basis for the broad utility of effective ER trainings like implementing intentions or cognitive-emotional training.

## Author Contributions

NK drafted the manuscript. All authors edited, revised, agreed to the final version of the manuscript, and contributed to the conception and conduction of the Research Topic.

## Conflict of Interest

The authors declare that the research was conducted in the absence of any commercial or financial relationships that could be construed as a potential conflict of interest.

## Publisher's Note

All claims expressed in this article are solely those of the authors and do not necessarily represent those of their affiliated organizations, or those of the publisher, the editors and the reviewers. Any product that may be evaluated in this article, or claim that may be made by its manufacturer, is not guaranteed or endorsed by the publisher.
